# Structures of variants of *Escherichia coli* flavodiiron-type nitric oxide reductase reveal changes in the di-iron site

**DOI:** 10.1107/S2059798326002214

**Published:** 2026-04-07

**Authors:** Patrícia T. Borges, Filipe Folgosa, Maria C. Martins, Guillaume Gotthard, Peter van der Linden, Philippe Carpentier, Miguel Teixeira, Carlos Frazão, Célia V. Romão

**Affiliations:** a ITQB NOVA, Instituto de Tecnologia Química e Biológica António Xavier Universidade Nova de Lisboa Avenida da República 2780-157Oeiras Portugal; b Swiss Light Source Paul Scherrer Institute Forschungsstrasse 111 5232Villigen Switzerland; c European Synchrotron Radiation Facility Avenue des Martyrs, CS 40220 38043Grenoble France; d ESRF, PSCM (Partnership for Soft Condensed Matter), 71 Avenue des Martyrs, 38000Grenoble, France; e Université Grenoble Alpes CEA CNRS, IRIG–LCBM UMR 5249, 17 Avenue des Martyrs, 38000Grenoble, France; University of Oxford, United Kingdom

**Keywords:** FDP, di-iron, crystallography, radiation damage, photoreduction

## Abstract

Flavodiiron proteins (FDPs) are NO and/or O_2_ reductases which contain a di-iron catalytic center. Interestingly, they exhibit different selectivities towards each one of these substrates, despite having the same ligands of the iron ions. *Escherichia coli* FDP is a selective NO reductase that protects this bacterium against nitric oxide by catalyzing two-electron reduction to the nontoxic N_2_O. Previously, based on kinetic studies, we explored the possible role of two amino acids located in the di-iron second coordination sphere, Lys53 and Tyr271, in modulation of the substrate selectivity of *Entamoeba histolytica* FDP, a selective O_2_ reductase. In this work, we replaced the structurally equivalent residues in *E. coli* FDP, Asp52 and Ser262, by those present in the O_2_-selective FDP and determined their crystal structures in both oxidized and reduced states. Furthermore, the molecular-substrate tunnels were experimentally identified using krypton pressurization of the crystals. The data obtained corroborated previous molecular-dynamics calculations on this FDP. The side chains of residues in both positions 52 and 262 of *E. coli* FDP variants and wild type are in the vicinity of the shorter intramolecular tunnel, which is suggested to be the exit route for the reaction products N_2_O and H_2_O. The *E. coli* FDP S262Y variant shows photoreduction of the di-iron center and partial loss of electron density in some of its coordinating ligands after X-ray exposure, and these effects are consistent with increased radiation sensitivity. The kinetic properties of the variants towards NO and O_2_ were not significantly different from the wild type, contrary to what was observed previously for *E. histolytica* FDP.

## Abstract 2

Flavodiiron proteins (FDPs) are di-iron enzymes that reduce NO and/or O_2_ with distinct substrate selectivity. We have produced *E. coli* FDP variants targeting second coordination-sphere residues and determined their structures. Although the kinetics remained unchanged, the *E. coli* FDP S262Y variant showed X-ray radiation-induced photoreduction, suggesting increased structural sensitivity without altered selectivity.

## 1. Introduction

Flavodiiron proteins (FDPs) are widespread in the three domains of life (Wasserfallen *et al.*, 1998 ▸; Smutná *et al.*, 2009 ▸; Di Matteo *et al.*, 2008 ▸; Sarti *et al.*, 2004 ▸; Gonçalves *et al.*, 2011 ▸; Peltier *et al.*, 2010 ▸; Allahverdiyeva *et al.*, 2011 ▸; Gomes *et al.*, 2002 ▸; Martins *et al.*, 2019 ▸). Due to their ability to reduce either oxygen and hydrogen peroxide, or nitric oxide, to nontoxic water and/or nitrous oxide, respectively, these proteins play an important role not only in microbial protection against oxidative and/or nitrosative stresses, but also in protection of the photosynthetic complexes of cyanobacteria and gymno­sperms (from algae to higher plants) in situations away from the natural redox balance (an excess of oxygen due to various environmental situations) (Gardner *et al.*, 2002 ▸; Kurtz, 2007 ▸; Martins *et al.*, 2019 ▸; Gomes *et al.*, 2002 ▸; Morvan *et al.*, 2021 ▸; Kint *et al.*, 2020 ▸; Romão *et al.*, 2016 ▸; Allahverdiyeva *et al.*, 2011 ▸). A classification of FDPs into nine classes was proposed based on the different structural domains that compose each protein (Folgosa *et al.*, 2018*a* ▸; Saraiva *et al.*, 2004 ▸; Martins *et al.*, 2019 ▸). All classes share the same two core domains that define this family of proteins: an N-terminal metallo-β-lactamase-like domain and a C-terminal flavodoxin-like domain (Frazão *et al.*, 2000 ▸; Silaghi-Dumitrescu *et al.*, 2005 ▸; Di Matteo *et al.*, 2008 ▸).

The first domain contains the catalytic site, an Fe–Fe center, in which the reduction of substrates occurs, whereas the second domain harbors a noncovalently bound flavin mononucleotide (FMN) which is responsible for donating electrons to the di-iron site. In all FDPs studied to date, the minimal quaternary arrangement is a ‘head-to-tail’ homodimer that results in a short distance (close to van der Waals contact, 3.5 Å) between the di-iron center of one monomer and the FMN of the opposing monomer, thus allowing efficient electron transfer. In fact, only two types of quaternary conformation have thus far been identified in catalytically competent enzymes, both in solution and in crystal structures, namely dimers or tetramers.

The catalytic site of most FDPs characterized thus far contains two Fe atoms coordinated by four histidines, two aspartates and one glutamate. A solvent bridge coordinating the two irons has been observed in the majority of FDPs. In both the *Escherichia coli* FDP (PDB entry 4d02; Romão *et al.*, 2016 ▸) and *Moorella thermoacetica* FDP structures (Silaghi-Dumitrescu *et al.*, 2003 ▸) it was assigned as a μ-hydroxo (μ-OH^−^) bridge. For the other FDP structures the exact chemical nature of this bridge, hydroxyl, aquo or oxo, has not been established (Di Matteo *et al.*, 2008 ▸; Frazão *et al.*, 2000 ▸; Silaghi-Dumitrescu *et al.*, 2005 ▸). Most of the characterized FDPs have a higher activity towards oxygen reduction (Frazão *et al.*, 2000 ▸; Silaghi-Dumitrescu *et al.*, 2005 ▸; Seedorf *et al.*, 2007 ▸; Di Matteo *et al.*, 2008 ▸; Folgosa *et al.*, 2018*b* ▸; Martins *et al.*, 2019 ▸, 2024 ▸).

In contrast, the FDP from *E. coli* (named flavorubredoxin due to the presence of a canonical rubredoxin domain after the C-terminal flavodoxin domain) is, so far, the only FDP with a clear preference for NO (Romão *et al.*, 2016 ▸; Gomes *et al.*, 2002 ▸; Gardner *et al.*, 2002 ▸). With the high conservation of the di-iron first coordination sphere among FDPs, irrespective of the substrate selectivity, the structural determinants for the observed differences in catalysis remain to be clarified. A comparison of model and crystal structures of FDPs with different selectivities has previously been performed (Gonçalves *et al.*, 2014 ▸). The O_2_-selective FDP from *Entamoeba histolytica* (*E. histolytica* FDP), which has a high amino-acid similarity to the O_2_-selective *Giardia intestinalis* FDP (PDB entry 2q9u), was compared with the NO-reducing *E. coli* FDP (PDB entry 4d02). Differences were observed at two positions within the di-iron second coordination sphere: Lys53 and Tyr271 in *E. histolytica* FDP are replaced by Asp52 and Ser262, respectively, in *E. coli* FDP. To assess the role of these two residues and with the aim of increasing the NO-reducing activity, the *E. histolytica* FDP variants K53D, Y271S and K53D/Y271S were constructed. The kinetic properties of the Y271S and K53D/Y271S variants indeed showed an increased activity towards NO and a higher sensitivity to O_2_, becoming inactive after multiple turnovers (Gonçalves *et al.*, 2014 ▸). These observations suggest a role of Tyr271 in the modulation of substrate specificity in FDPs. Therefore, as an attempt to demonstrate a symmetric behavior, the reverse experiment, *i.e.* substitution of the equivalent residues in *E. coli* FDP by those in *E. histolytica* FDP, was performed. To obtain an enzyme similar to *E. histolytica* FDP, D52K, S262Y and D52K/S262Y variants of *E. coli* FDP were introduced in a construct lacking the C-terminal rubredoxin domain, herein named FDP-ΔRd. These variants were characterized biochemically and kinetically and their crystal structures were determined. Furthermore, to experimentally determine substrate/product intramolecular tunnels, which were previously hypothesized by molecular dynamics and structural analysis for the *Desulfovibrio gigas* and *E. coli*FDPs (Victor *et al.*, 2009 ▸), we used krypton high-pressurization of FDP-ΔRd D52K crystals to map the internal gas network in the enzyme.

## 2. Materials and methods

### 2.1. Protein expression and purification

Expression and purification of wild-type *E. coli* FDP-ΔRd were performed as described previously (Vicente & Teixeira, 2005 ▸; Romão *et al.*, 2016 ▸). The site-directed variants (from residues 1 to 412) were cloned into pET-24a(+) plasmids using codons optimized for *E. coli*; sequencing was performed to ensure that the mutations were correctly inserted into the nucleotide sequence. All plasmids were obtained from GenScript, USA. *E. coli* BL21(DE3) Gold cells were transformed with the plasmid and plated at 37°C onto LB plates containing kanamycin (30 µg ml^−1^). These cells were used to inoculate a pre-inoculum grown in LB at 37°C overnight. A 2 l culture in minimal medium (M9) supplemented with 100 µ*M* FeSO_4_·7H_2_O at 37°C and 140 rev min^−1^ was inoculated with 5% of the pre-inoculum. Growth was induced at an optical density (600 nm) of 0.4 with 100 µ*M* isopropyl β-d-1-thio­galactopyranoside (IPTG) at 30°C. After 7 h of induction, the cells were harvested by centrifugation at 10 000*g* for 10 min at 4°C. The cells were then suspended in 20 m*M* Tris–HCl pH 7.5, disrupted in a French press at 6.9 MPa and ultracentrifuged at 100 000*g* at 4°C for 1 h. The soluble fraction was dialysed at 4°C overnight against 20 m*M* Tris–HCl pH 7.5 with 2.5%(*v*/*v*) glycerol.

Purification of *E. coli* FDP-ΔRd and FDP-ΔRd S262Y was performed in two consecutive steps using an ÄKTAprime system (Cytiva) at 4°C, as described previously (Vicente & Teixeira, 2005 ▸). The soluble fraction was firstly loaded onto an anionic Q Sepharose Fast Flow column equilibrated with 20 m*M* Tris–HCl pH 7.5, 5%(*v*/*v*) glycerol and eluted using a linear gradient from 0 to 1 *M* NaCl. Fractions containing the flavin UV–visible fingerprint (absorption bands with maxima at 375 and 455 nm) and a low Abs (280 nm)/Abs (455 nm) ratio were eluted at ∼250 m*M* NaCl and were pooled. The second purification step consisted of size-exclusion chromatography on a Superdex 200 column (XK26/60) equilibrated with 20 m*M* Tris–HCl pH 7.5, 250 m*M* NaCl, 5%(*v*/*v*) glycerol. Protein purity was confirmed by SDS–PAGE and the fractions were pooled and concentrated to 15 mg ml^−1^.

A similar protocol was used to purify the different *E. coli* FDP-ΔRd variants for the catalytic activity assays, for which 18% of glycerol was present in the buffers and only the anion-exchange purification step was performed.

The expression and purification procedures for the rubredoxin domain (Rd-D) of *E. coli* FDP and its redox partner NADH:flavorubredoxin oxidoreductase (NROR) was performed as described previously (Vicente & Teixeira, 2005 ▸).

### 2.2. Protein, metal and flavin quantification

Pure protein samples to be used in the catalytic assays were quantified using the bicinchoninic acid kit (Thermo) with bovine serum albumin as a standard (Smith *et al.*, 1985 ▸). Iron content was determined by the phenanthroline colorimetric method (Pyenson & Tracy, 1945 ▸): briefly, protein samples were incubated for 15 min with 1 *M* HCl and for a further 30 min with 10% trichloroacetic acid at room temperature (25°C) and centrifuged at 5000*g* for 20 min. Samples were then incubated with 10% hydroxylamine and 0.3% 1,10-phenanthroline. The absorbance was measured at 510 nm with ɛ_510 nm_ = 11.2 m*M*^−1^ cm^−1^. The flavin content was determined by thermal denaturation of each protein sample at 100°C for 20 min, followed by centrifugation at 5000*g* for 15 min. The absorbance spectrum of the protein-free supernatant was measured and the flavin content was quantified using ɛ_446 nm_ = 11.1 m*M*^−1^ cm^−1^.

### 2.3. Spectroscopic methods

UV–visible spectra were obtained using a PerkinElmer Lambda 35 spectrophotometer. Electron paramagnetic resonance (EPR) spectroscopy was performed using a Bruker EMX spectrometer equipped with an Oxford Instruments ESR-900 continuous-flow helium cryostat and a perpendicular mode rectangular cavity. Protein samples were prepared aerobically at final concentrations of 100 µ*M*. Partially reduced samples were prepared anaerobically inside a Coy glove box by incubating the protein samples with one equivalent of menadiol. EPR spectra were simulated using the program *SpinCount* (Petasis & Hendrich, 2015 ▸).

### 2.4. Protein quaternary-structure determination

The quaternary structure of the proteins was determined by size-exclusion chromatography. Protein samples were loaded separately at 4°C onto a 25 ml Superdex S200 10/300 GL column (GE) previously equilibrated with 20 m*M* Tris–HCl pH 7.5 containing 18% glycerol and 150 m*M* NaCl. As standards, a mixture containing aldolase (molecular weight 158 kDa), ferritin (440 kDa), conalbumin (75 kDa), carbonic anhydrase (29 kDa) and cytochrome *c* (12.4 kDa), with dextran blue (molecular weight 2000 kDa) as a void volume marker, was used.

### 2.5. Protein crystallization

Crystallization conditions were screened with a nanodrop crystallization robot (Cartesian, Genomic Solutions) using the sitting-drop vapor-diffusion method with round-bottom Greiner 96-well CrystalQuick plates (Greiner Bio-One). The *E. coli* FDP-ΔRd D52K, S262Y and D52K/S262Y variants formed crystals within two days at 20°C in condition A9 of Structure Screen I and II (Molecular Dimensions) consisting of 0.1 *M* sodium citrate pH 5.6, 20%(*w*/*v*) PEG 4000, 20%(*v*/*v*) 2-propanol using a drop consisting of 0.1 µl protein solution (15 mg ml^−1^) and 0.2 µl reservoir solution. Following this crystallization hit, microlitre-scale optimization proceeded using the hanging-drop vapor-diffusion method in XRL 24-well crystallization plates (Molecular Dimensions). In these scaled-up experiments different ranges of conditions were tested, namely the PEG 4000 (15–28%) and 2-propanol (15–25%) concentrations and also the pH of the sodium citrate solution (4.5–8.0). After 2–3 days, orange thin-plate crystals reached maximal lengths of 0.1–0.2 mm in 0.1 *M* sodium citrate pH 5.6, 20–23%(*w*/*v*) PEG 4000, 23–25%(*v*/*v*) 2-propanol when using 1 µl protein solution (15 mg ml^−1^) and 2 µl reservoir solution.

Crystals were cryoprotected with the reservoir solution supplemented with 10%(*v*/*v*) glycerol prior to flash-cooling in liquid nitrogen. In order to obtain crystal structures of the reduced state of the enzyme, an excess of sodium dithionite powder was added to the cryoprotected crystals. Bleaching of the orange color provided confirmation that the proteins were reduced prior to flash-cooling in liquid nitrogen. Crystals of the as-isolated state were considered to be in the oxidized state and are labeled ‘oxi’, while those that were chemically reduced are labeled ‘red’.

### 2.6. Pressurization of crystals with krypton

Krypton pressurization of *E. coli* FDP-ΔRd D52K crystals was performed to localize protein hydrophobic tunnels (Carpentier *et al.*, 2020 ▸). Crystals of this variant were chosen based on their reduced sensitivity to X-ray-induced radiation damage. Crystals were mounted on a specific high-pressure support developed at the European Synchrotron Radiation Facility (ESRF) High Pressure Freezing Laboratory for Macromolecular Crystallography (HPMX) (van der Linden *et al.*, 2014 ▸; Lafumat *et al.*, 2016 ▸; Carpentier *et al.*, 2024 ▸) and placed in the krypton pressure cell. They were pressurized at 10 MPa at ambient temperature for 5 min to allow the noble-gas atoms to diffuse into the crystal lattice and label the proteins. Next, they were flash-cooled to cryogenic temperature while still under pressure in order to cryo-trap the krypton-derivative state. The samples were then depressurized at cryogenic temperature to preserve this derivative state. Finally, they were transferred into cryo-caps in liquid nitrogen and placed into a sample-changer puck for diffraction data collection. The crystal of *E. coli* FDP-ΔRd D52K pressurized with krypton was labeled as ‘Kr’.

### 2.7. X-ray diffraction, crystal structure determination and refinement

Diffraction data of *E. coli* FDP-ΔRd variants were measured at 100 K on synchrotron beamlines (Table 1 ▸). Mesh-scan analysis was essential to obtain good-quality diffracting data from crystals of *E. coli* FDP-ΔRd variants since, although reflections were visible, they could not be properly indexed, probably due to the multiple stacked thin-plate crystals. Diffraction spots were indexed, integrated, scaled and the final amplitudes were calculated using *XDS* (Kabsch, 2010 ▸). Data-collection details and processing statistics are listed in Table 1 ▸. The distribution of the Matthews coefficient (Matthews, 1968 ▸; Kantardjieff & Rupp, 2003 ▸) indicated a high probability that there were two *E. coli* FDP-ΔRd molecules in the asymmetric unit. The structure of the S262Y_oxi_ variant was solved by molecular replacement with *Phaser* (McCoy *et al.*, 2007 ▸) within the *Phenix* suite (Liebschner *et al.*, 2019 ▸), using the coordinates of the native *E. coli* FDP structure (PDB entry 4d02; Romão *et al.*, 2016 ▸) as the search model. As all of the present *E. coli* FDP-ΔRd variants were isomorphous with the S262Y_oxi_ crystal, the two monomers in the asymmetric unit were refined using *phenix.refine* (Adams *et al.*, 2010 ▸; Afonine *et al.*, 2012 ▸; Terwilliger *et al.*, 2008 ▸) using initial rigid-body refinement followed by atomic position and isotropic atomic displacement parameter (ADP) refinement. The *TLSMD* server (https://skuld.bmsc.washington.edu/~tlsmd; Painter & Merritt, 2006 ▸) was used to define polypeptide chain regions for translation, libration and screw (TLS) refinement of anisotropic ADPs. Cycles of iterative model inspection and editing against σ_A_-weighted electron-density maps with *Coot* (Emsley *et al.*, 2010 ▸) were alternated with model refinement using *phenix.refine*. Although the refinement included standard stereochemistry libraries (Engh & Huber, 1991 ▸), interatomic distances involving iron centers were refined without target restraints. For μ-hydroxo bridge and Kr atoms, refinement cycles were performed with successively reduced occupancies until their ADPs reached values similar to those of neighboring atoms. Solvent water molecules were automatically assigned from σ_A_ difference map peaks to neighboring hydrogen-bonding acceptors/donors within distances of 2.45–3.40 Å. Other solvent molecules were identified through comparison of their shapes against electron-density blobs, as well as by comparing their refined ADPs with those of neighboring atoms. Refinement and model edition iterations were performed until the *R*_work_ and *R*_free_ values converged, before calculation of the final *R*_cryst_ using the available diffraction data. In the last refinement cycle containing all experimental data, the weighting factors for stereochemistry and ADPs were manually set in order to obtain bond and angle root-mean-square deviations (r.m.s.d.s) similar to those in the previous cycle when *R*_free_ had been used to set the refinement strategy.

The stereochemistry of the refined structures was analyzed with *MolProbity* (Chen *et al.*, 2010 ▸). Analysis of molecular tunnels was performed with *MOLE* 2.0 (Sehnal *et al.*, 2013 ▸) and *PyMOL* (DeLano, 2002 ▸; Schrödinger). Figures showing structural models were prepared with *PyMOL*. For the sake of simplicity, structural comparisons of the variant structures involve only their *A* chain. Refinement statistics are presented in Table 1 ▸.

### 2.8. UV–visible absorption of protein crystals and calculation of absorbed X-ray doses

Offline UV–visible absorption spectra of crystals were recorded at 100 K on the microspectrophotometer of the *ic*OS Lab at ESRF (Royant *et al.*, 2007 ▸; von Stetten *et al.*, 2015 ▸) before and after X-ray irradiation exposure, using a high-sensitivity fixed-grating QE65Pro spectrophotometer with a back-thinned CCD detector (Ocean Optics, Dunedin, Florida, USA) and an Oxford Cryostream (Oxford Cryosystems Ltd, Oxford, UK). Fig. 5 was prepared using the *ic*OS toolbox (Caramello *et al.*, 2025 ▸).

The X-ray doses absorbed by crystals of *E. coli* FDP and *E. coli* FDP-ΔRd S262Y were determined with *RADDOSE*-3*D* (Zeldin *et al.*, 2013 ▸) using the diffraction-weighted dose metric (DWD; Table 2 ▸).

### 2.9. Amperometric measurements of O_2_ and NO reductase activities

The O_2_ and NO reductase activities were measured amperometrically with modified Clark-type electrodes selective for O_2_ (Oxygraph-2K, Oroboros Instruments, Innsbruck, Austria) or NO (ISO-NOP, World Precision Instruments, Sarasota, Florida, USA). The assays were performed in 50 m*M* Tris–HCl pH 7.5 containing 18%(*v*/*v*) glycerol. The O_2_ reductase activity was evaluated at 25°C in air-equilibrated buffer (∼250 µ*M* O_2_) in the presence of 5 m*M* NADH, 1.35 µ*M* Rd-D and 0.5 µ*M* NROR. The reaction was initiated by the addition of 4 µ*M* FDP-ΔRd or its variants. Assays were performed in the presence of catalase and superoxide dismutase (640 and 240 n*M*, respectively) to eliminate any species resulting from incomplete reduction of O_2_. The NO reductase activity was determined under anaerobic conditions in 50 m*M* Tris–HCl pH 7.5 containing 18%(*v*/*v*) glycerol in the presence of an O_2_-scavenging system (10 m*M* glucose, 375 n*M* glucose oxidase and 750 n*M* catalase). Sequential additions of NO (up to 12 µ*M*) were followed by the addition of 5 m*M* NADH, 0.3 µ*M* Rd-D and 0.7 µ*M* NROR. The reaction was initiated by the addition of 0.2 µ*M* FDP-ΔRd or its variants. Stock solutions of 1.91 m*M* NO were prepared by saturating a degassed solution of 50 m*M* Tris–HCl pH 7.5 containing 18%(*v*/*v*) glycerol buffer in a rubber-sealed capped flask with pure NO gas (Air Liquide) at 1 atm on ice: gaseous NO was flushed through a 5 m*M* KOH trap to remove higher *N*-oxides and a second trap with deionized water to remove aerosols. After this, the solution was allowed to equilibrate at room temperature.

For all of the assays, the turnover rates (s^−1^) were calculated by subtracting the experimental slope (µ*M* s^−1^) before and after the addition of each enzyme, divided by the protein concentration (in µ*M*). These values were then normalized considering the iron content of each enzyme, similarly to as described previously (Martins *et al.*, 2021 ▸).

## 3. Results

### 3.1. Structure determination and quality

As mentioned above, all proteins were crystallized in the as-isolated state (oxidized state) and in the reduced state by chemical reduction with a solution of sodium dithionite. The crystal structures were refined at resolutions ranging from 1.85 to 2.54 Å (Table 1 ▸). The polypeptide chain of each variant was traced in the electron density within the residue ranges 1–401 for FDP-ΔRd D52K_oxi_ and 2–401 or 2–402 for the remaining variants. The electron-density maps allowed the localization of two Fe atoms and one FMN molecule per monomer in all crystallographic structures, each refined with full occupancy. The μ-hydroxo bridge was refined with full occupancy, except for the FDP-ΔRd S262Y_oxi_, S262Y_red_ and D52K/S262Y_red_ structures, which were refined with occupancies ranging from 0.42 to 0.91. Most of the residues are within the most favored region of the Ramachandran plot (Table 1 ▸).

Final models included one FMN cofactor, two Fe atoms, one μ-hydroxo bridge and one dioxygen molecule for almost all of the structures obtained, except for chain *B* of *E. coli* FDP-ΔRd D52K_red_, which presents a 2-propanol molecule (IPA). The krypton-pressurization experiment led to the presence of three Kr atoms in each chain of *E. coli* FDP-ΔRd D52K_Kr_.

### 3.2. Overall structure of *E. coli* FDP-ΔRd variants

**Figure 1:**
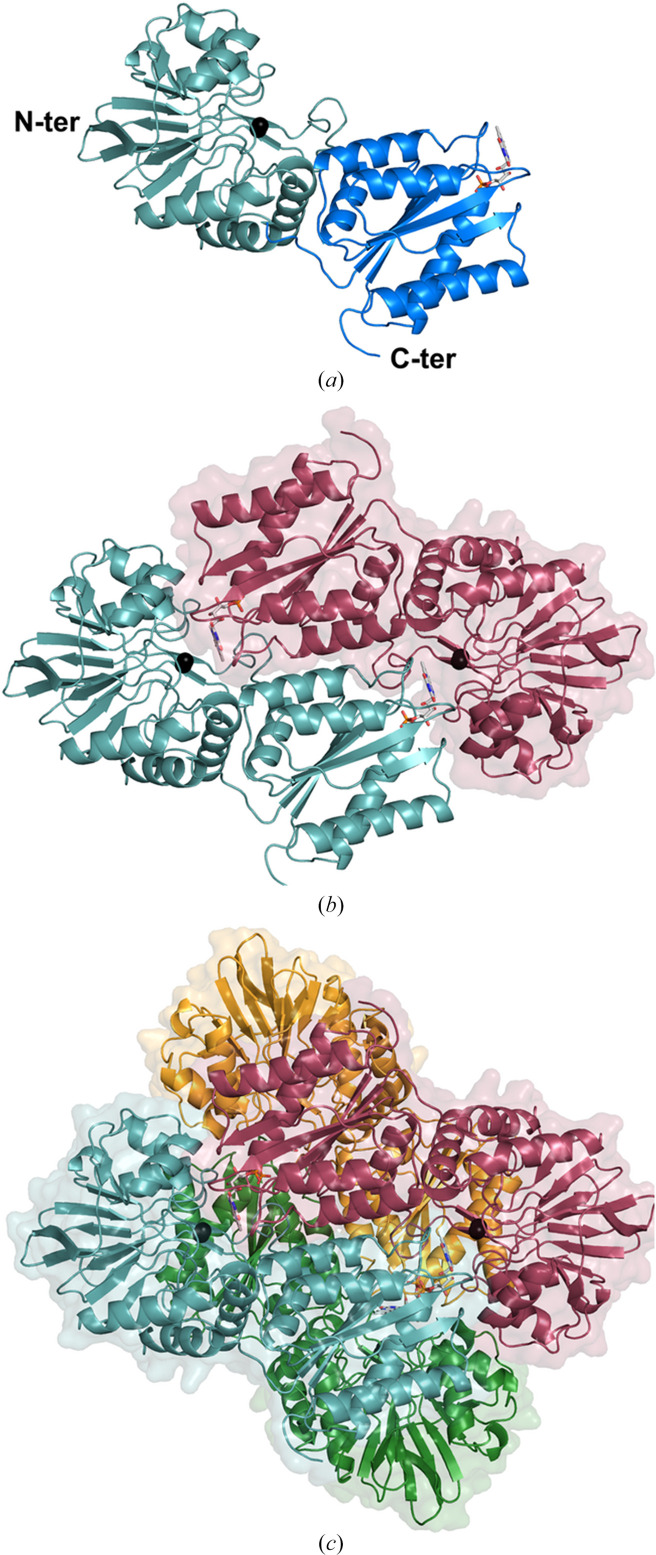
Overall structure of *E. coli* FDP-ΔRd D52K. (*a*) Cartoon representation of the monomer composed of two structural domains: an N-terminal metallo-β-lactamase-like domain (cyan) and a C-terminal flavodoxin-like domain (blue). (*b*) Cartoon representation of the ‘head-to-tail’ dimer and transparent solvent-accessible surface of one of the monomers, with one chain represented in dark red and the other in cyan. (*c*) Cartoon representation and transparent solvent-accessible surface of the tetramer, where the chains are colored dark red, cyan, green and orange. Fe atoms are represented as black spheres and FMN is shown with C, O, N and P atoms as gray, red, blue and orange sticks, respectively.

The overall fold of the several *E. coli* FDP-ΔRd variants in different oxidation states did not show significant differences. Although the asymmetric units of the crystal unit cells include two independent monomers, the characteristic ‘head-to-tail’ dimer of FDP is not visible in the asymmetric unit. Each monomer of the *E. coli* FDP-ΔRd variant, as in other FDPs, contains two structural domains: a metallo-β-lactamase-like domain located in the N-terminal region (residues 1–246) and a flavodoxin-like domain in the C-terminal region (residues 247–401) (Fig. 1 ▸*a*). Nevertheless, the FDP dimers become evident when applying the twofold-rotation crystal symmetry, which produces the corresponding ‘head-to-tail’ opposing partner (Fig. 1 ▸*b*) for each monomer. The final sets of four chains correspond to a dimer of dimers with pseudo-222 point-group symmetry (Fig. 1 ▸*c*), in agreement with the size-exclusion chromatography data and similar to the native *E. coli* FDP. Superimposition of the independent molecules from *E. coli* FDP-ΔRd variants showed a low r.m.s.d. between C^α^ atoms (0.11–0.23 Å). Their superposition with other FDP structures (from *Giardia intestinalis*, *Thermotoga maritima*, *Methano­thermobacter marburgensis*, *Moorella thermoacetica* and *Desulfovibrio gigas*) also showed similar three-dimensional arrangements with overall C^α^ r.m.s.d.s within 1.38–1.93 Å.

### 3.3. Structural features in the di-iron site

**Figure 2:**
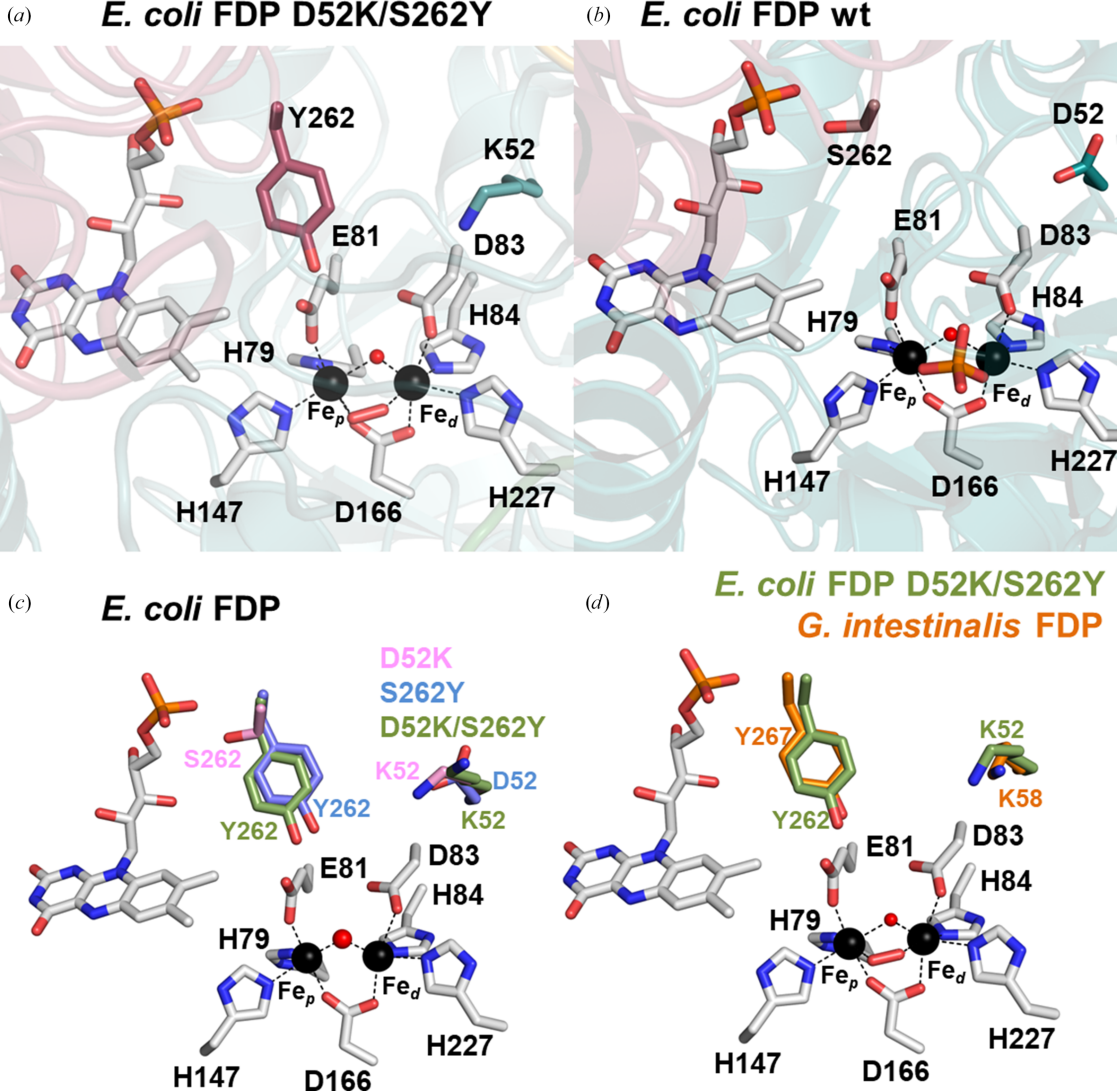
Di-iron catalytic site of FDPs. (*a*) Representation of the Lys52 and Tyr262 residues from the di-iron second coordination sphere of *E. coli* FDP-ΔRd D52K/S262Y. Cartoon representation of the monomers, colored in dark red and cyan. (*b*) Representation of the Asp52 and Ser262 residues from the di-iron second coordination sphere of *E. coli* FDP (PDB entry 4d02). Cartoon representation of the monomers, colored in dark red and cyan. (*c*) Superposition of the di-iron second coordination spheres from *E. coli* FDP-ΔRd D52K (pink), S262Y (blue) and D52K/S262Y (green). (*d*) Superposition of the di-iron second coordination sphere from *E. coli* FDP-ΔRd D52K/S262Y (green) with *G. intestinalis* FDP (PDB entry 2q9u; orange). Fe atoms are represented as black spheres. The amino-acid residues and FMN are shown as sticks with C atoms in gray, N atoms in blue, O atoms in red and P atoms in orange. The μ-hydroxo bridge is shown as a red sphere.

In all FDP-ΔRd variants the metal ligands are conserved: four imidazole N atoms and three O atoms from carboxylate residues; Fe_p_ (the Fe proximal to FMN) is coordinated by Glu81 OE1, Asp166 OD2, His147 NE2 and His79 NE2, and Fe_d_ (the Fe distal to FMN) is coordinated by Asp83 OD2, Asp166 OD1, His84 NE2 and His227 NE2 (Fig. 2 ▸*a*). This coordination is similar to that previously described for *E. coli* FDP, as well as most other FDPs (Fig. 2 ▸*b*; Romão *et al.*, 2016 ▸). The interatomic distances that the Fe atoms establish with the amino-acid ligands and other species are listed in Table 3 ▸.

**Figure 3:**
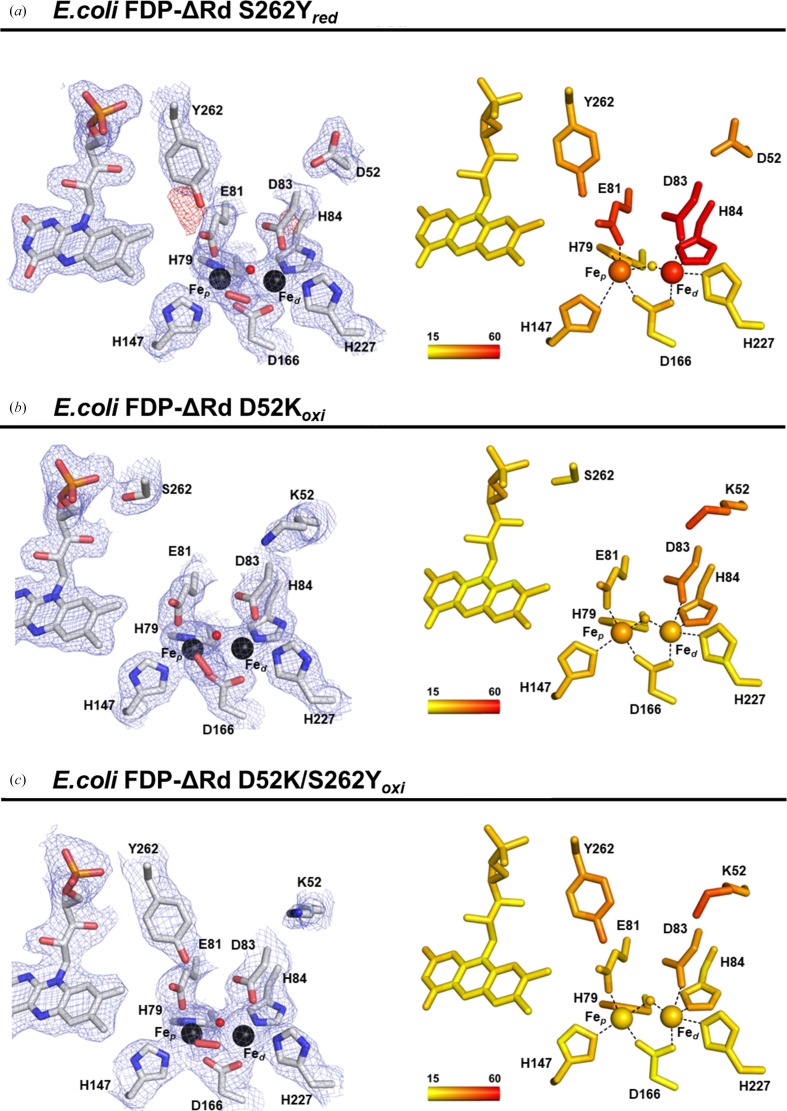
Active site of *E. coli* FDP-ΔRd variants. Left panels: structure representation of the di-iron site and mutated residues of *E. coli* FDP-ΔRd S262Y_red_ (*a*), D52K_oxi_ (*b*) and D52K/S262Y_oxi_ (*c*) with 2*m*|*F*_o_| − *D*|*F*_c_| electron-density map in blue, contoured at 1.5σ, and *m*|*F*_o_| − *D*|*F*_c_| electron-density map in red, contoured at 3.0σ. Iron ligands and FMN are shown as sticks with C atoms in gray, N atoms in blue, O atoms in red and P atoms in orange. Right panels: the same representation as in the left panels but with the amino-acid residues, Fe atoms, solvent bridge and FMN colored in a ramp from yellow to red according to the 〈ADP〉 ranging from 15 to 60 Å^2^.

As mentioned above, two residues of the di-iron site second coordination sphere, Asp52 and Ser262, were mutated to lysine and tyrosine, respectively (Fig. 2 ▸*c*). However, while Asp52 is located ∼8 Å from Fe_d_ in the same monomer, the Ser262 residue is ∼10 Å from Fe_p_ and belongs to the ‘head-to-tail’ opposing monomer. The Lys52 and Tyr262 residues are in the same structural positions as Lys58 and Tyr267 from *G. intestinalis* FDP (Fig. 2 ▸*d*). The di-iron active site is similar within the *E. coli* FDP-ΔRd variant structures (Fig. 3 ▸, Supplementary Fig. S1 and Table 3 ▸), with the exception of S262Y_red_ and S262Y_oxi_ that present higher ADP values for the metal ligands Glu81, Asp83 and His84 (41–56 Å^2^; Fig. 3 ▸*a* and Supplementary Fig. S1*a*) relative to other variant structures (35–43 Å^2^; Figs. 3 ▸*b* and 3 ▸*c* and Supplementary Figs. S1*b* and S1*c*).

### 3.4. FMN binding site

As in other FDPs, the FMN present in the *E. coli* FDP-ΔRd variants is located at the interface between the two monomers of the ‘head-to-tail’ homodimer. The phosphorylated part of FMN neighbors the residues at position 262, while the methyl group of the isoalloxazine ring is close to the di-iron center, at van der Waals distance from the iron ligand Glu81.

### 3.5. Molecular tunnels

**Figure 4:**
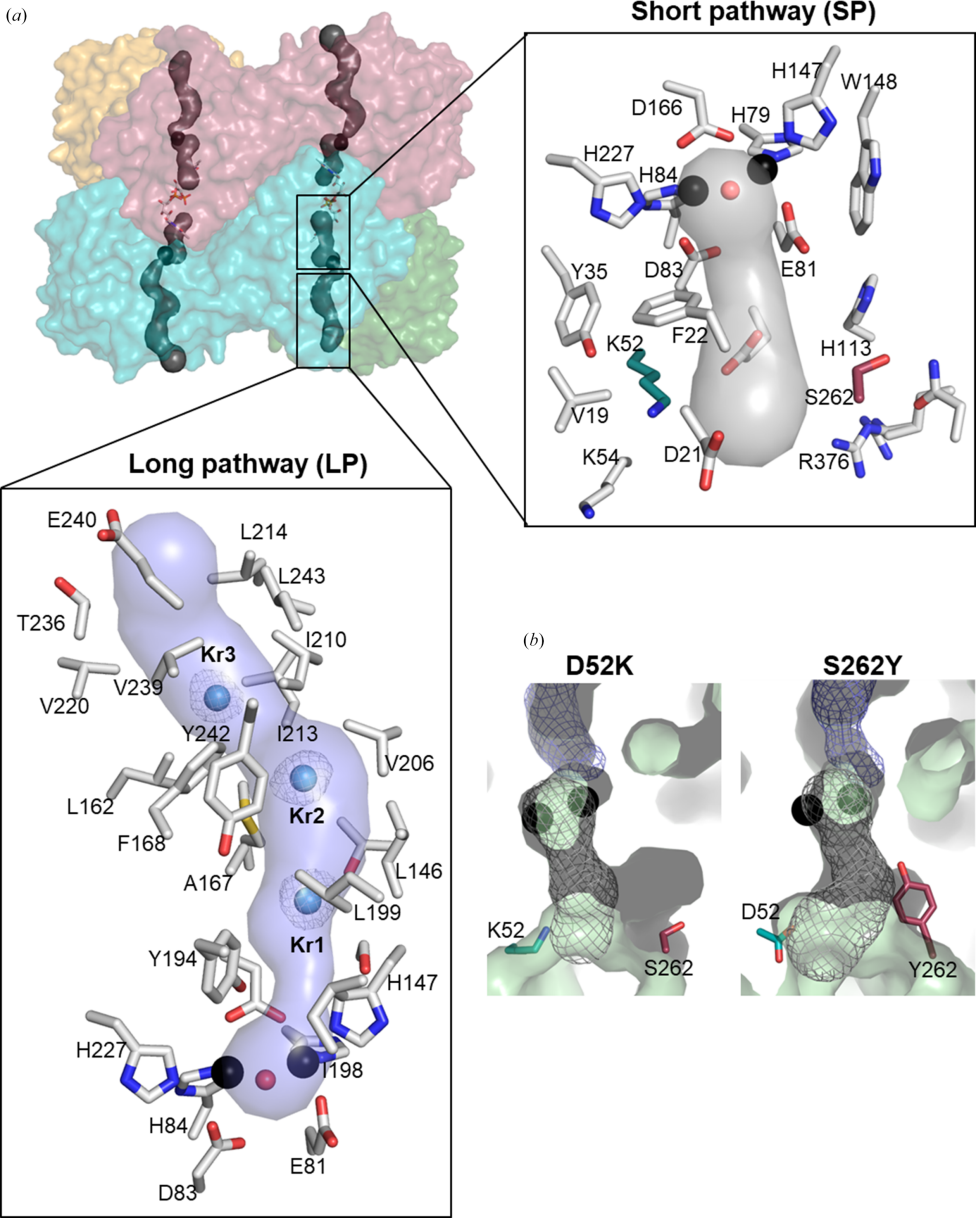
*E. coli* FDP-ΔRd D52K and S262Y molecular tunnels. (*a*) Transparent solvent-accessible surface of the *E. coli* FDP-ΔRd D52K tetramer, colored as in Fig. 1 ▸, showing both tunnel pathways. The long pathway (LP) connects the external surface of the protein with the di-iron catalytic site. Kr atoms (Kr1–Kr3) localized in the LP are shown as blue spheres and the 2*m*|*F*_o_| − *D*|*F*_c_| electron-density map is contoured at 1.5σ (gray mesh). The short pathway (SP) is in the opposite direction to the long pathway, connecting the di-iron site with the interior of the tetramer. The Lys52 and Ser262 residues are located near the SP and are shown with C atoms in cyan and dark red, respectively. Residues lining the SP and LP are represented by sticks with C atoms in gray, O atoms in red and N atoms in blue. (*b*) Long and short pathways from *E. coli* FDP-ΔRd D52K and S262Y variants. The residues at position 52 (cyan) and 262 (dark red) are located near the SP. The LP and SP are shown as a blue and gray mesh, respectively.

As for the *E. coli* FDP (Romão *et al.*, 2016 ▸; Fig. 2 ▸*b*), the molecular surface of the *E. coli* FDP-ΔRd D52K variant, defined with a 1.4 Å rolling probe, shows one tunnel that crosses the active-site pocket (Fig. 4 ▸), where dioxygen molecules were observed in both the oxidized and reduced forms. These dioxygen molecules are also found in the chemically reduced form of *E. coli* FDP (PDB entry 5lld) and are replaced by a phosphate molecule in the oxidized *E. coli* FDP (PDB entry 4d02; Romão *et al.*, 2016 ▸). The tunnel can be divided into two sections: (i) a longer pathway, ∼23 Å long and with a diameter of ∼3.4–12.0 Å, that is lined mainly by apolar atoms (Fig. 4 ▸*a*) and (ii) a shorter ∼9 Å pathway, with a diameter of ∼2.8–5.7 Å, showing a lower fraction of apolar atoms (Fig. 4 ▸*a*). The two pathways run in opposite directions, with the longer pathway reaching the external protein surface, while the shorter pathway reaches the solvent medium at the hollow interior of the tetramer. Based on crystallographic structures and molecular-dynamics simulations, it was previously proposed (Romão *et al.*, 2016 ▸) that the longer pathway could be the main entrance for the apolar substrates (O_2_ and/or NO), while exit of the more polar products of the reactions (N_2_O and H_2_O) could occur through the shorter pathway. In both the *E. coli* FDP-ΔRd S262Y and D52K/S262Y variants, the Tyr262 residue constricts the 7 Å wide side gallery that connects the short pathway to the external surface of the protein (Fig. 4 ▸*b*).

Noble gases such as krypton or xenon have been used in the investigation of hydrophobic paths, and since they have high atomic numbers they lead to high anomalous scattering (Schiltz *et al.*, 2003 ▸; Murray & Barber, 2007 ▸; Murray *et al.*, 2008 ▸; Gabdulkhakov *et al.*, 2009 ▸). Moreover, krypton has a similar van der Waals radius to those of molecular oxygen and nitric oxide, which makes this a convenient gas to probe for possible O_2_ and NO tunnels, and it has been used to identify a molecular tunnel in the FDP family member FprA from *Methanothermobacter marburgensis* (Engilberge *et al.*, 2020 ▸). To confirm the localization of the observed tunnel structures, D52K variant crystals were pressurized with krypton gas prior to diffraction data collection (D52K_Kr_). In the longer tunnel of the *E. coli* FDP-ΔRd D52K_Kr_ crystal structure, three density blobs were observed and were initially assigned as putative water molecules. However, Fourier difference maps revealed that these density features exhibited relatively high electron density comparable to that of the di-iron atoms, even at a low contour level (∼4σ). The corresponding density blobs were not present in the D52K structure. Moreover, they displayed significantly lower ADPs compared with neighboring atoms, 3–14 Å^2^ versus 27–46 Å^2^, respectively, and were therefore assigned as Kr atoms (Fig. 4 ▸*a*). As the refined Kr atoms showed rather high ADP values, refinement cycles were used to successively reduce their occupancies until their ADPs reached values similar to neighboring atoms. Final Kr-atom occupancies ranged between 0.4 and 0.6. All Kr atoms are located within the long pathway: Kr1 is near the active site, Kr2 is positioned in the intermediate region and Kr3 is close to the molecular outer surface.

### 3.6. Photoreduction

**Figure 5:**
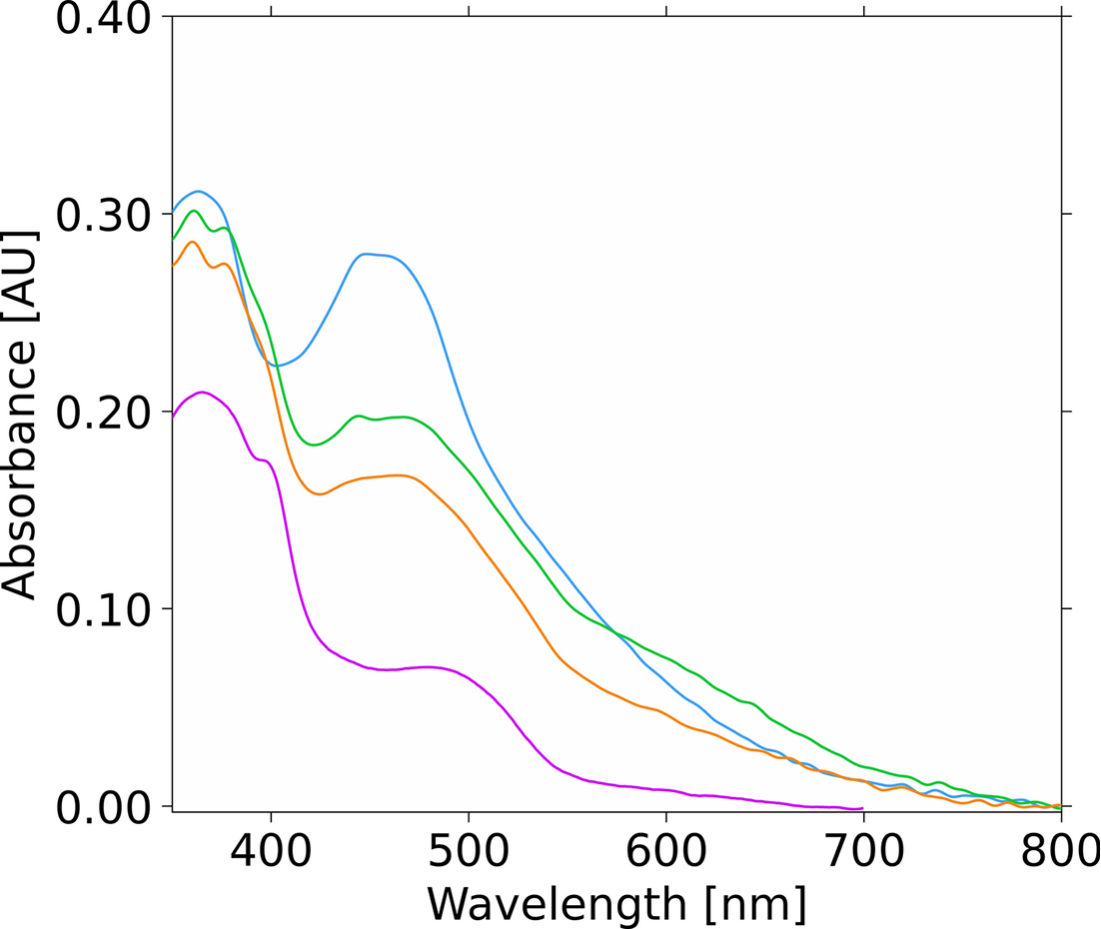
UV–Vis absorption spectra of a crystal of *E. coli* FDP-ΔRd S262Y. Blue, crystal before X-ray exposure; green, crystal exposed to X-rays during dataset collection (dose 1.22 MGy); orange, the same crystal position as represented in green but exposed to more X-ray radiation (dose 2.92 MGy); for reference, in pink, spectrum of an FDP in solution with the FMN semiquinone state.

UV–Vis arbsorption spectra of FDP-ΔRd S262Y crystals were measured at 100 K before and after exposure to X-rays (von Stetten *et al.*, 2015 ▸). A total of five crystals were tested and a similar result was obtained for all of them. Before data collection, the typical FMN UV–visible absorption band is observable with two maxima at 375 and 455 nm, identical to the spectrum of the protein in solution and compatible with the fully oxidized state of the cofactor (Fig. 5 ▸; Gomes *et al.*, 2000 ▸). However, after collecting a 180° dataset (corresponding to a dose ranging from 0.56 to 1.49 MGy, depending on the crystal), a decrease in the typical FMN absorption band at 455 nm was observed as well as a slight shift of the band at 375 nm (Fig. 5 ▸). Subtracting the crystal spectrum before exposure to the X-rays from that obtained after irradiation, the resulting spectrum resembles a typical FMN anionic semiquinone (Fig. 5 ▸). This indicates formation of the FMN semiquinone species upon exposure to a low dose of radiation. It is interesting to observe that a further data collection at the same position on the crystal, with an additional 180° rotation (corresponding to a further dose of 2.92 MGy), shows that this broad band is still present in the after–before difference spectrum. The observed change only affected the region that was irradiated by X-rays.

### 3.7. Catalytic activities

In order to understand the effect of the mutations on the catalytic activities, *E. coli* FDP-ΔRd and its variants were purified to homogeneity and were determined to be homotetramers in solution by size-exclusion chromatography, similar to the wild-type *E. coli* FDP. The flavin content was determined to be ∼0.7 FMN per monomer for all of the variants (Table 4 ▸), whereas the iron content was ∼1.2, which are lower values than the expected contents for the proteins under study (two Fe atoms and one flavin per monomer), as observed previously for proteins from the same family (Martins *et al.*, 2024 ▸; Folgosa *et al.*, 2018*b* ▸).

All variants exhibit UV–visible spectra, in the oxidized state, that are almost identical to the FDP-ΔRd protein, with maxima at 372 and 460 nm, as a result of the presence of the FMN cofactor. The key distinction between these and *E. coli* FDP is, as expected, the absence of a contribution from the rubredoxin center. As observed for all FDPs (see, for example, Martins *et al.*, 2024 ▸, 2025 ▸; Folgosa *et al.*, 2018*b* ▸), the di-iron center is not observable by UV–visible spectroscopy due to its low molar absorptivity and to the overlapping contribution of the flavin cofactor in the absorption spectrum. Therefore, the di-iron was studied by EPR spectroscopy upon substoichiometric reduction with one equivalent of menadiol. The EPR spectra of the mixed-valence state of the variants was almost the same as those of the wild-type protein, indicating that the mutations did not affect the geometry of the catalytic center. The *g* values obtained by spectral simulation are presented in Table 4 ▸.

The O_2_ and NO reductase activities of *E. coli* FDP-ΔRd and its variants were determined amperometrically by using modified Clark-type electrodes selective for each gas (Gomes *et al.*, 2000 ▸, 2002 ▸). Because the target proteins only have the two core domains, and NROR is not able to reduce FDPs directly, the catalytic assays were performed in the presence of Rd-D and NROR to ensure electron transfer between the electron donor NADH and the catalytic domain of the *E. coli* FDP-ΔRd and its variants (Table 4 ▸). *E. coli* FDP-ΔRd showed a low O_2_ reduction activity, with a turnover rate of 0.68 ± 0.07 s^−1^, when compared with a rate of 2.2 s^−1^ for the full-length protein (Gomes *et al.*, 2000 ▸; Martins *et al.*, 2021 ▸). For the D52K/S262Y, S262Y and D52K variants we obtained turnovers of 0.60 ± 0.12, 0.39 ± 0.07 and 0.22 ± 0.04 s^−1^, respectively. We consider that the differences observed are negligible due to the very low activities determined. Nevertheless, the oxygen-consumption profile is similar for all four variants, *i.e.* there were no inhibitory effects observed, in contrast to the observations made for the *E. histolytica* FDP variants (Gonçalves *et al.*, 2014 ▸; Supplementary Fig. S2).

Similarly, the NO reduction rates determined were, within experimental errors, of the same magnitude (Table 4 ▸ and Supplementary Fig. S3). As also observed for O_2_ reduction, the NO-consumption profile was similar for all the variants.

## 4. Discussion

FDPs are a family of proteins that afford protection from oxygen and/or NO in bacteria, archaea and some eukarya. To date, the molecular determinants that affect substrate selectivity in these proteins remain unknown. However, mutation studies on the O_2_-selective *E. histolytica* FDP indicated that the Y271S mutation, in the di-iron second coordination sphere, contributes to an increase in the activity with nitric oxide relative to the native protein (Gonçalves *et al.*, 2014 ▸) but, most importantly, showed that Tyr271 has a protective role in the reaction with oxygen, preventing inactivation of the enzyme by blocking the formation of reaction intermediates that could lead to enzyme inactivation. In order to unravel the molecular determinants underlying substrate selectivity in *E. coli* FDP, single and double variants were generated, aiming to convert this NO reductase into an O_2_ reductase. The design of these point mutations was based on the abovementioned kinetic studies on the *E. histolytica* FDP O_2_ reductase.

Crystal structures were determined for three truncated *E. coli* FDP-ΔRd variants: the single and double variants D52K, S262Y and D52K/S262Y. All structures revealed a homotetrameric assembly (Fig. 1 ▸) consistent with size-exclusion chromatography results and in agreement with previous reports that FDPs generally form either dimers or tetramers under *in vitro* conditions (see, for example, Martins *et al.*, 2019 ▸). Structural comparisons showed that the mutated residues Lys52 and Tyr262 in *E. coli* FDP-ΔRd are in equivalent structural positions when compared with the corresponding residues, Lys58 and Tyr267, of the *G. intestinalis* FDP O_2_ reductase, which in turn is very similar to the *E. histolytica* enzyme (Fig. 2 ▸).

The di-iron active site is conserved in all *E. coli* FDP-ΔRd single- and double-variant structures. However, in the reduced form of the S262Y variant (S262Y_red_), the di-iron ligands Glu81, Asp83 and His84 have higher ADP values when compared with the same residues in the D52K and D52K/S262Y variants, indicating greater structural flexibility or disorder in this region (Fig. 3 ▸*a*).

In the *E. coli* FDP-ΔRd structures presented here, the Fe_p_–Fe_d_ distances are consistently longer in the chemically reduced state compared with those in the oxidized state, with the exception of the S262Y variant (Table 3 ▸). For instance, in the D52K variant this distance increased from 3.3 Å (oxidized) to 3.7 Å (reduced). Notably, all of these proteins exhibit a μ-hydroxo bridge at the di-iron site. It is interesting to compare these results with the previous published *E. coli* FDP structure (Romão *et al.*, 2016 ▸), where the Fe_p_–Fe_d_ distance was reported to decrease upon reduction, from 3.5 Å in the oxidized state to 3.2 Å in the chemically reduced state. However, no μ-hydroxo bridge was observed in that structure, which could explain the shorter distance between the Fe atoms compared with the structures presented here. These observations suggest that the presence or absence of a μ-hydroxo bridge may significantly influence the Fe–Fe distance and the structural response to redox changes, but this remains to be confirmed and warrants further investigation.

As mentioned above, the S262Y structures are exceptions with respect to the changes in iron–iron distances between the oxidized and reduced states. The oxidized S262Y crystal exhibited an Fe_p_–Fe_d_ distance of approximately 3.6 Å, similar to the chemically reduced state, suggesting that this variant may be particularly sensitive to X-ray-induced photoreduction (Fig. 5 ▸). In fact, X-ray exposure of S262Y crystals led to the formation of an anionic semiquinone spectrum characteristic of FMN in most FDPs, indicating photoreduction of the redox-active centers such as FMN. In the native *E. coli* FDP, the FMN cofactor has reduction potentials of −40 and −130 mV, while the di-iron center has potentials of −20 and −90 mV (Vicente & Teixeira, 2005 ▸). Therefore, it is likely that the di-iron site becomes partially reduced upon irradiation, potentially leading to the mixed-valence state in this variant.

Tyrosine/tryptophan chains are known to play a key role in protecting oxidoreductases from radical-induced damage by acting as escape routes to the external protein surface (Gray & Winkler, 2015 ▸; Winkler & Gray, 2015 ▸), as proposed for *E. coli* FDP (Romão *et al.*, 2016 ▸). In the S262Y and D52K/S262Y variants, an additional aromatic residue (Tyr262) is introduced relative to native FDP that connects the di-iron site to the external surface of the protein. Analysis of the solvent-accessible surface area of Tyr262 in FDP-ΔRd S262Y revealed a greater exposure (152.4 Å^2^) compared with the D52K/S262Y double variant (129.1 Å^2^). This difference appears to result from conformational variations in the Glu82 ligand side chain located near the Tyr262 residue. In the D52K/S262Y variant structure Glu82 is oriented closer to Tyr262, limiting its exposure, whereas in the S262Y variant Glu82 adopts a more distant conformation, thus increasing the solvent accessibility of Tyr262. Difference Fourier maps for *E. coli* FDP-ΔRd S262Y_red_ and and S262Y_oxi_ showed negative electron densities near the ligand Asp83 C^β^ and the nearby Tyr262 OH (Fig. 3 ▸*a* and Supplementary Fig. S1*a*). The altered densities observed in the active site and its vicinity result from X-ray exposure; however, further studies are required to confirm the possibility of X-ray-induced damage. Nevertheless, it is known that water photolysis occurs during X-ray irradiation, which produces extremely reactive free-radical species in macromolecular protein crystals (Southworth-Davies & Garman, 2007 ▸), even at cryogenic temperatures, and so radiation damage can occur due to the high beam intensities available at synchrotron beamlines (Burmeister, 2000 ▸; Garman, 2003 ▸).

In order to understand the nature of these structural changes in *E. coli* FDP-ΔRd mutants, we estimated the absorbed radiation dose for the various datasets (Table 2 ▸). The diffraction-weighted dose (DWD) values were clearly below the experimentally accepted dose limit of 30 MGy in macromolecular crystallography (Owen *et al.*, 2006 ▸), but there are reports of radiation damage to protein crystals with doses of 1.0–1.5 MGy (Corbett *et al.*, 2007 ▸; Adam *et al.*, 2004 ▸) and, in particular, metal reduction takes place at much lower doses. Although both the D52K and D52K/S262Y crystals were exposed to a higher absorbed dose relative to those of the two redox states of S262Y, their active site and surrounding neighborhood did not show any relevant structural changes, which suggests that the observed crystallographic anomalies may be related to the replacement of a serine by a tyrosine residue at position 262. To exclude the possibility that the observed electron-density anomalies derived from fortuitous protein mishandling, a new batch of the *E. coli* FDP-ΔRd S262Y protein was produced, purified and crystallized. Crystals were obtained under the same conditions, diffraction datasets were collected and the corresponding structures were refined with similar resolutions, 1.90–2.20 Å. Both crystal structures showed similarly altered electron density at the active site and neighboring residues. Another possible explanation would be a Tyr aromatic ring displacement, as hasbeen proposed for some protein structures, for example myrosinase (Bury *et al.*, 2017 ▸). In the *E. coli* FDP-ΔRd S262Y crystals, refinement of alternate conformations of the Tyr ring with different occupancies was attempted. In all attempts the negative electron density observed near Tyr262 OH persisted.

To complement structural data, molecular-dynamics simulations are often employed. In the case of *E. coli* FDP, this methodology was fundamental in identifying diffusion pathways for both oxygen and nitric oxide (Romão *et al.*, 2016 ▸). Molecular tunnel analysis of its structure revealed a long pathway connecting the di-iron active site to the external surface. Supporting this model, our present crystallographic data identified three Kr atoms along this pathway after gas pressurization (Fig. 4 ▸*a*), confirming the route predicted by the previous molecular-dynamics simulations (Victor *et al.*, 2009 ▸; Romão *et al.*, 2016 ▸). Interestingly, comparative studies across FDPs from *D. gigas* (O_2_/NO reductase), *E. coli* (NO reductase) and *G. intestinalis* (O_2_ reductase) revealed that despite differences in substrate selectivity, these proteins exhibit similar diffusion properties for both diatomic molecules (Romão *et al.*, 2016 ▸). Additionally, a shorter tunnel was proposed to connect the active site to an internal void in the tetramer assembly. This route, which has lower apolarity and weaker affinity for O_2_ or NO molecules compared with the long path, may serve as an exit tunnel for reaction products (Victor *et al.*, 2009 ▸; Romão *et al.*, 2016 ▸). Residues at positions 52 and 262 in *E. coli* FDP are located near this short tunnel. Notably, the Tyr262 residue introduced in both the single and double variants narrows a side gallery that connects this tunnel to the external surface of the protein (Fig. 4 ▸*b*). It is also noteworthy that the long pathway tunnel in which Kr atoms were observed in *E. coli* FDP-ΔRd D52K_Kr_ is comparable to that found in the FDP family member from *M. marburgensis*, F_420_H_2_-oxidase (FprA), where six Kr atoms were detected (Engilberge *et al.*, 2020 ▸).

Structural analysis of the *E. coli* FDP-ΔRd variants did not reveal any evidence that could explain the substrate selectivity towards NO, and therefore we have complemented our structural data with kinetic studies. Nevertheless, contrary to what was observed with *E. histolytica* FDP (Gonçalves *et al.*, 2014 ▸), kinetic characterization of the NO and O_2_ reduction performed by FDP-ΔRd or its variants did not show significant differences, which points to low relevance of these amino acids for substrate selectivity in *E. coli* FDP (Table 4 ▸).

## 5. Conclusion

In this study, we explored possible molecular determinants involved in substrate selectivity of *E. coli* FDP by generating and structurally characterizing single and double variants, D52K, S262Y and D52K/S262Y, designed based on comparative analysis with the O_2_-reducing *E. histolytica* FDP. Structural comparisons revealed that the introduced mutations resulted in local rearrangements near the di-iron site and the short diffusion pathway. In the *E. coli* FDP-ΔRd S262Y variant, X-ray exposure induces partial photoreduction of the di-iron cluster and slight changes of its ligand environment. Despite these structural alterations, kinetic assays demonstrated that none of the variants showed a significant shift in substrate preference toward O_2_. In fact, all variants showed O_2_- and NO-reductase activity profiles identical to those of FDP-ΔRd. The low values for the activities towards both substrates of all four proteins, *i.e.* including the wild-type construct FDP-ΔRd, are likely to be attributable to the absence of the rubredoxin domain, as demonstrated for other FDPs, such as those from *Clostridioides difficile* and *Syntrophomonas wolfei* (Folgosa *et al.*, 2018*b* ▸; Martins *et al.*, 2024 ▸). Additionally, the identification of Kr atoms along the long tunnel supports the proposed presence of structured diffusion channels in an NO-selective FDP. Altogether, our findings indicate that unlike in *E. histolytica*, mutations at positions 52 and 262 in *E. coli* FDP are insufficient to redirect the substrate preference, highlighting the very complex and enzyme-specific nature of substrate selectivity. Further investigations integrating the rubredoxin domain and exploring additional molecular determinants are necessary to fully understand and potentially reprogram substrate specificity in *E. coli* FDP. Remarkably, mutations of the enzyme from *E. coli* in a stretch of amino acids conserved among all FDPs of the same enzyme class led to drastic changes in the NO-reducing activity, but the molecular basis for the observed effects remains to be understood (Martins *et al.*, 2021 ▸).

## Supplementary Material

PDB reference: *Escherichia coli* FDP, D52K mutant, oxidized state, 7r0f

PDB reference: D52K mutant, reduced state, 7r1h

PDB reference: S262Y mutant, oxidized state, 7r2o

PDB reference: S262Y mutant, reduced state, 7r1j

PDB reference: D52K/S262Y mutant, oxidized state, 7r2p

PDB reference: D52K/S262Y mutant, reduced state, 7r2r

PDB reference: D52K mutant, pressurized with krypton gas, 7r2s

Supplementary Figures. DOI: 10.1107/S2059798326002214/gm5120sup1.pdf

## Figures and Tables

**Table 1:** Data-collection and refinement statistics for *E. coli* FDP-ΔRd mutants Values in parentheses are for the highest resolution shell.

	D52K_oxi_	D52K_red_	S262Y_oxi_	S262Y_red_	D52K/S262Y_oxi_	D52K/S262Y_red_	D52K_Kr_
Data collection							
Beamline	ID-29, ESRF	P14, PETRA III	ID-29, ESRF	ID-29, ESRF	ID-23.2, ESRF	ID-23.2, ESRF	ID-23.1, ESRF
Wavelength (Å)	0.9762	0.9763	1.0000	0.9762	0.8726	0.8726	0.9724
Space group	*I*2	*I*2	*I*2	*I*2	*I*2	*I*2	*I*2
*a*, *b*, *c* (Å)	89.1, 64.1, 146.7	88.9, 64.9, 146.7	89.4, 64.4, 147.3	89.2, 64.2, 146.6	89.1, 64.9, 147.3	89.6, 64.7, 147.9	89.1, 64.1, 146.7
α, β, γ (°)	90, 91.16, 90	90, 91.53, 90	90, 91.09, 90	90, 91.04, 90	90, 91.60, 90	90, 91.42, 90	90, 91.16, 90
Resolution (Å)	76.81–1.98 (2.07–1.98)	75.18–1.95 (2.05–1.95)	75.83–1.90 (2.00–1.90)	76.80–1.90 (2.00–1.90)	75.39–2.54 (2.64–2.54)	77.44–2.20 (2.29–2.20)	52.33–2.20 (2.30–2.20)
No. of observations	193300 (29494)	137447 (20721)	129422 (18166)	221835 (35709)	114331 (16785)	217362 (32264)	107641 (17131)
Unique reflections	56529 (8571)	60345 (8881)	57099 (8802)	65086 (10367)	27589 (4039)	42726 (6547)	41495 (6564)
Completeness (%)	95.9 (90.5)	98.2 (89.3)	96.8 (88.5)	98.7 (97.9)	97.8 (89.3)	98.7 (94.1)	96.2 (94.5)
Multiplicity	3.4 (3.4)	2.3 (2.3)	2.3 (2.1)	3.4 (3.4)	4.1 (4.1)	5.1 (4.9)	2.6 (2.6)
Mosaicity (°)	0.2	0.2	0.6	0.2	0.4	0.2	0.3
CC_1/2_	0.98 (0.67)	0.96 (0.69)	0.99 (0.52)	0.99 (0.63)	0.97 (0.52)	0.98 (0.58)	0.98 (0.74)
*R*_sym_ (%)	13.8 (53.5)	12.5 (53.6)	10.9 (58.8)	12.9 (48.6)	20.0 (67.3)	19.5 (67.5)	12.9 (43.5)
*R*_meas_ (%)	18.3 (96.7)	17.1 (70.5)	13.7 (86.3)	16.1 (68.8)	26.3 (129.1)	25.0 (119.2)	18.0 (72.6)
*R*_p.i.m._ (%)	8.8 (33.6)	8.3 (35.7)	7.5 (49.5)	8.2 (30.5)	11.1 (37.2)	9.5 (32.6)	9.3 (30.5)
〈*I*/σ(*I*)〉	5.8 (1.7)	5.5 (1.4)	5.2 (1.3)	6.9 (1.9)	5.2 (1.2)	6.3 (1.3)	5.7 (1.8)
Wilson *B* factor (Å^2^)	24	19	21	19	35	24	22
*V*_M_ (Å^3^ Da^−1^)	2.28	2.30	2.28	2.28	2.31	2.33	2.28
Estimated solvent content (%)	45.9	46.5	45.9	46.1	46.8	47.2	45.9
Refinement statistics							
*R* factor (%)	20.5	18.7	19.9	18.5	20.7	19.2	23.7
*R*_work_ (%)	21.4	20.1	21.7	19.6	22.0	20.9	25.4
*R*_free_ (%)	24.0	24.0	23.5	22.4	23.7	26.2	29.5
R.m.s.d., bond lengths (Å)	0.010	0.009	0.018	0.013	0.012	0.008	0.013
R.m.s.d., bond angles (°)	0.580	0.650	0.560	0.695	0.405	0.561	0.572
Average chain *B* factor† (Å^2^)	27, 29	20, 23	25, 28	21, 23	35, 36	27, 29	26, 28
Average FMN *B* factor† (Å^2^)	21, 20	15, 16	23, 21	18, 16	26, 29	21, 22	16, 21
Average Fe *B* factor† (Å^2^)	28–32, 30–33	33–34, 32–34	39–48, 40–44	27–31, 29–32	28–38, 31–42	22–23, 22–23	29–36, 33–35
No. of residues	401	400	400	400	400	400	400
No. of solvent waters	518	725	783	791	122	365	381
Ramachandran plot							
Residues in favored regions (%)	96.2	97.8	96.5	96.4	96.7	97.5	97.9
Residues in allowed regions (%)	3.8	2.2	3.5	3.6	3.3	2.5	2.1
Residues in disallowed regions (%)	0	0	0	0	0	0	0
PDB code	7r0f	7r1h	7r2o	7r1j	7r2p	7r2r	7r2s

† The average *B*-factor values for chains *A* and *B* are indicated, separated by a comma.

**Table 2:** Calculated X-ray diffraction-weighted absorbed doses for cuboid-shaped crystals

	D52K_oxi_	D52K_red_	S262Y_oxi_	S262Y_red_	D52K/S262Y_oxi_	D52K/S262Y_red_
Dimensions (µm)	100, 100, 100	100, 100, 100	200, 100, 50	200, 100, 50	100, 100, 100	100, 100, 100
Gaussian beam type, full-width and half-maximum (µm)	10, 10	10, 10	30, 50	20, 20	10, 10	10, 10
Beam flux (photons s^−1^)	1.4 × 10^11^	1.3 × 10^11^	6.3 × 10^11^	1.7 × 10^11^	1.1 × 10^11^	1.2 × 10^11^
Energy (keV)	14.2	12.7	12.4	12.7	14.2	14.2
Exposure time (s)	125.3	129.6	29.6	24.0	118.9	136.5
Dose (MGy)	7.50	6.20	1.07	0.81	6.87	9.25

**Table 3:** Distance (Å) between Fe atoms (Fe_p_ and Fe_d_) and atoms from metal-ligand residues Values in parentheses correspond to the equivalent distances in chain *B*.

Distance (Å)	D52K_oxi_	D52K_red_	S262Y_oxi_	S262Y_red_	D52K/S262Y_oxi_	D52K/S262Y_red_	D52K_Kr_
Fe_p_–His79 NE2	2.4 (2.5)	2.4 (2.4)	2.7 (2.9)	2.4 (2.5)	2.5 (2.5)	2.4 (2.4)	2.4 (2.4)
Fe_p_–Glu81 OE1	2.0 (2.0)	2.1 (2.1)	1.9 (1.9)	1.7 (1.8)	2.0 (2.0)	1.9 (2.0)	1.9 (1.9)
Fe_p_–His147 NE2	2.2 (2.3)	2.2 (2.3)	2.6 (2.8)	2.4 (2.5)	2.4 (2.4)	2.4 (2.3)	2.3 (2.4)
Fe_p_–Asp166 OD2	2.1 (2.1)	2.0 (2.1)	2.1 (2.2)	2.1 (2.1)	2.1 (2.2)	2.0 (2.2)	2.1 (2.2)
Fe_p_–μOH	1.8 (1.9)	1.9 (2.1)	2.1 (2.1)	2.2 (2.4)	2.0 (2.0)	2.0 (2.2)	1.9 (2.0)
Fe_p_–O_2_ O	2.8 (3.0)	3.0 (3.0)	3.1 (3.2)	3.2 (3.3)	3.0 (3.1)	2.9 (3.2)	3.2 (3.2)
Fe_d_–Asp83 OD2	2.4 (2.5)	2.4 (2.4)	2.3 (2.3)	2.4 (2.4)	2.5 (2.6)	2.3 (2.4)	2.4 (2.5)
Fe_d_–His84 NE2	2.0 (2.1)	2.0 (2.1)	2.2 (2.2)	2.1 (2.1)	2.1 (2.2)	2.0 (2.1)	2.1 (2.1)
Fe_d_–Asp166 OD1	2.3 (2.3)	2.2 (2.3)	2.5 (2.7)	2.3 (2.4)	2.3 (2.3)	2.4 (2.4)	2.4 (2.5)
Fe_d_–His227 NE2	2.2 (2.2)	2.1 (2.2)	2.2 (2.2)	2.2 (2.3)	2.2 (2.1)	2.1 (2.2)	2.2 (2.1)
Fe_d_–μOH	1.9 (1.9)	2.0 (2.2)	1.7 (1.7)	1.8 (1.8)	1.7 (1.8)	2.0 (2.1)	1.9 (2.0)
Fe_d_–O_2_ O	3.0 (3.0)	2.7 (2.8)	2.5 (2.7)	2.6 (2.9)	2.8 (2.9)	2.4 (2.4)	3.2 (3.1)
Fe_p_–Fe_d_	3.3 (3.4)	3.7 (3.7)	3.6 (3.6)	3.6 (3.7)	3.4 (3.4)	3.6 (3.6)	3.4 (3.4)

**Table 4:** Biochemical and spectroscopic characterization of FDP-ΔRd and mutants The *g* values resulted from EPR spectral simulations.

	Cofactor analysis		Enzymatic activity		
Protein	Iron/protein	Flavin/protein	O_2_ reduction (s^−1^)	NO reduction (s^−1^)	EPR (*g* values of the mixed-valence sate)
FDP-ΔRd	1.15 ± 0.09	0.71 ± 0.03	0.68 ± 0.07	0.46 ± 0.05	1.94, 1.80, 1.74
D52K	1.20 ± 0.20	0.59 ± 0.06	0.22 ± 0.04	0.21 ± 0.09	1.95, 1.78, 1.72
S262Y	1.03 ± 0.18	0.64 ± 0.03	0.39 ± 0.07	0.42 ± 0.11	1.95, 1.80, 1.74
D52K/S262Y	1.24 ± 0.21	0.75 ± 0.08	0.60 ± 0.12	0.25 ± 0.06	1.96, 1.78, 1.74
