# Supplementary material for: Structures of variants of Escherichia coli flavodiiron-type nitric oxide reductase reveal changes in the di-iron site
Source: Acta Crystallogr D Struct Biol. 2026 Apr 7;82(Pt 5):457–70. doi: 10.1107/S2059798326002214 (PMC13133992; doi:10.1107/S2059798326002214)
Supplement: Supplementary file 1 [file d-82-00457-sup1.pdf]

Acta Cryst D

STRUCTURAL
BIOLOGY

Volume 82 (2026)

Supporting information for article:

Structures of variants of *Escherichia coli* flavodiiron-type nitric oxide reductase reveal changes in the di-iron site

Patrícia T. Borges, Filipe Folgosa, Maria C. Martins, Guillaume Gotthard, Peter van der Linden, Philippe Carpentier, Miguel Teixeira, Carlos Frazão and Célia V. Romão

Figure S1

A. *E.coli* FDP-ΔRd S262Y$_{oxi}$

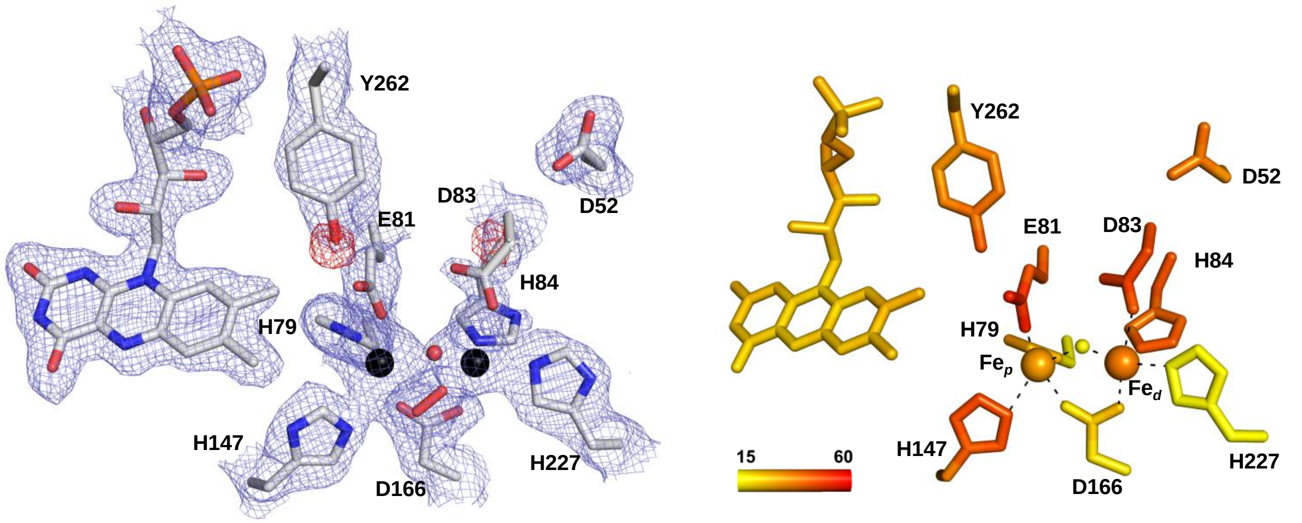

B. *E.coli* FDP-ΔRd D52K$_{red}$

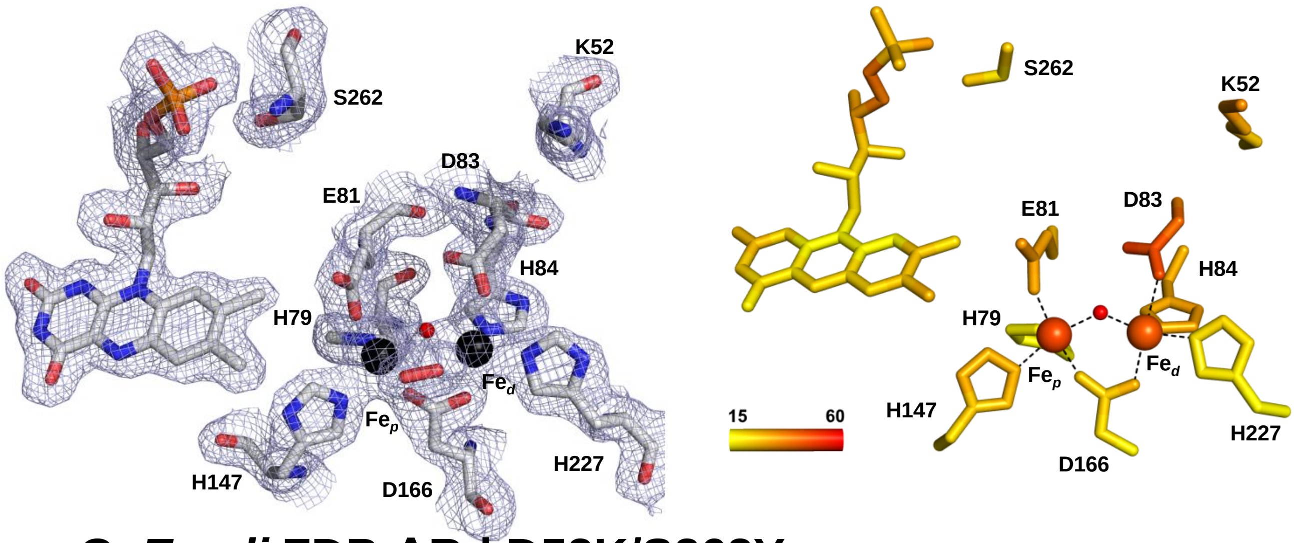

C. *E.coli* FDP-ΔRd D52K/S262Y$_{red}$

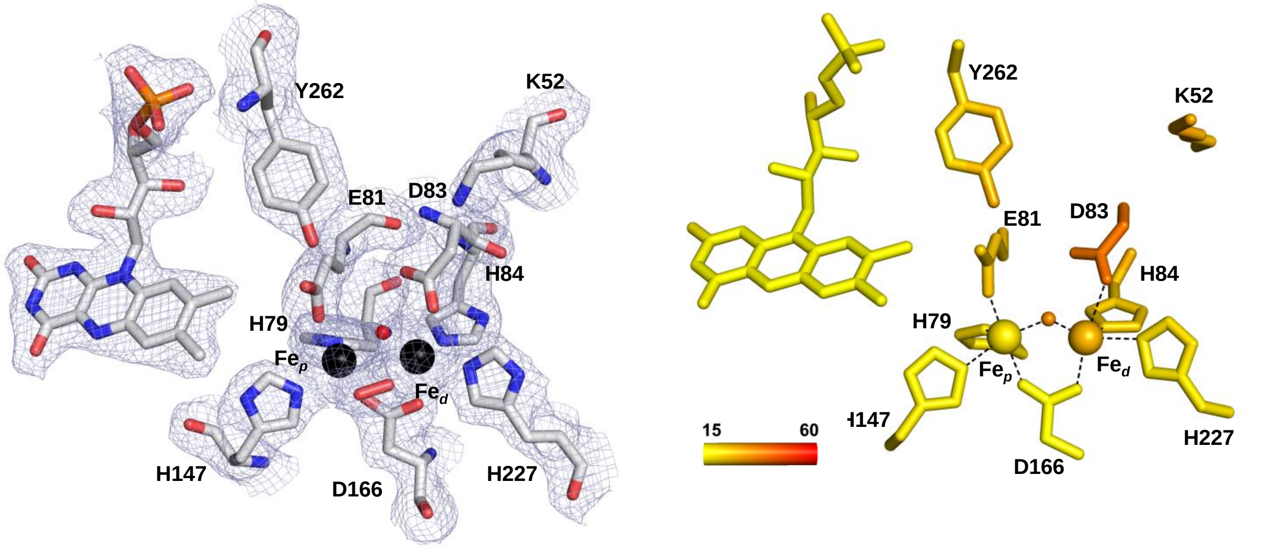

Figure S1 - *E. coli* FDP-ΔRd variants active site.

A, B, C - left panel. Structure representation of the di-iron site and mutated residues of *E. coli* FDP-ΔRd S262Y$_{oxi}$, D52K$_{red}$ and D52K/S262Y$_{red}$, respectively, with map electron density 2m|Fo|-D|Fc| in blue and m|Fo|-D|Fc| in red. Iron ligands and FMN are shown as sticks with carbon atoms in grey, nitrogen in blue, oxygen in red and phosphorous atoms in orange.

A, B, C - right panel. Same representation as in left panels but the amino acid residues, iron atoms, solvent bridge and FMN are colored ramping from yellow to red according to the <*a.d.p.*>s ranging from 15 Å^2 to 60 Å^2.

Figure S2

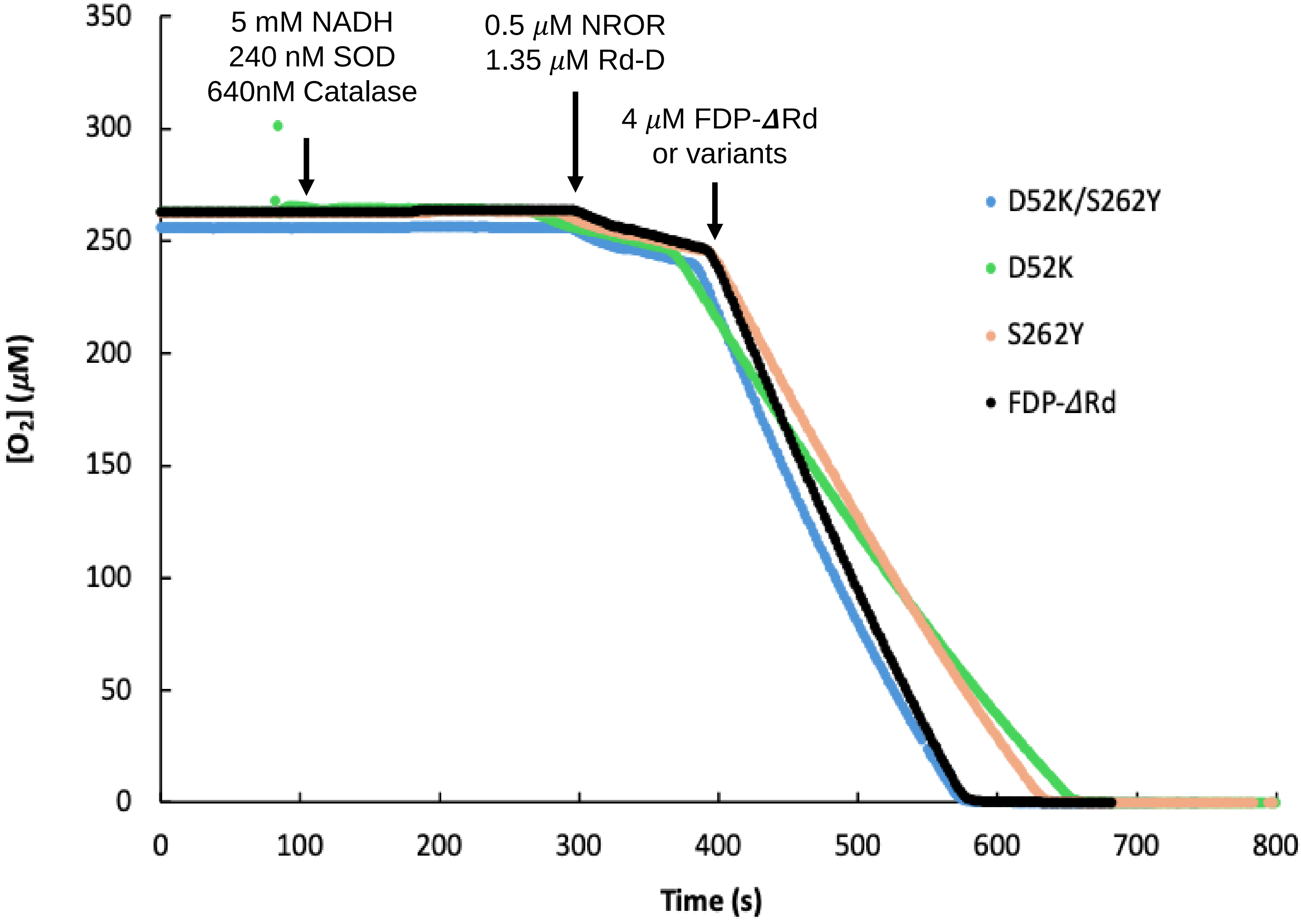

Figure S2 - O_2 reduction activities of *E. coli* FDP-**Δ**Rd (black line) and variants (D52K/S262Y in blue, D52K in green and S262Y in orange). Assays were performed in a modified Clark type electrode with air equilibrated buffer (50 mM Tris-HCl pH 7.5 containing 18% glycerol), with 5mM NADH, 240 nM SOD and 640nM Catalase. Protein concentrations were 0.5 μM of *E. coli* NROR, 1.35 μM of rubredoxin domain (Rd-D) of *E. coli* FDP and 4 μM of *E. coli* FDP-**Δ**Rd and variants.

Figure S3

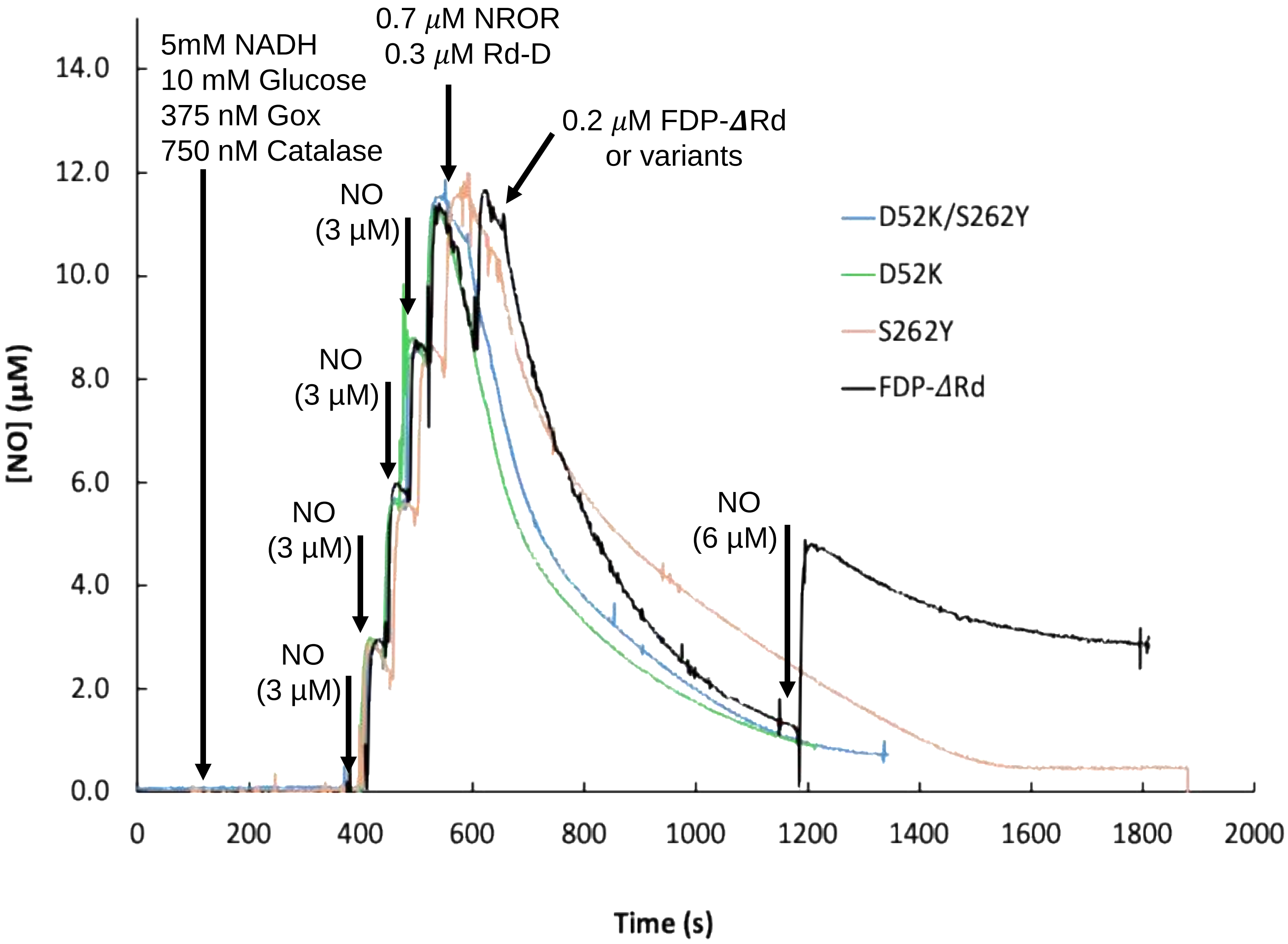

Figure S3 - NO reduction activities of *E. coli* FDP-**Δ**Rd (black line) and variants (D52K/S262Y in blue, D52K in green and S262Y in orange). Assays were performed anaerobically with a modified Clark type electrode in 50 mM Tris-HCl pH 7.5 containing 18% glycerol buffer. The reaction mixture also contained 5mM NADH, 10 mM glucose, 375 nM glucose oxidase and 750 nM catalase. Protein concentrations were 0.7 μM of *E. coli* NROR, 0.3 μM of rubredoxin domain (Rd-D) of *E. coli* FDP and 0.2 μM of *E. coli* FDP-**Δ**Rd and variants.
